# Breast Cancer Cytochromes P450: Chemopreventive and/or Therapeutic Targets for Naturally Occurring Phytochemicals

**DOI:** 10.3390/molecules30153079

**Published:** 2025-07-23

**Authors:** Hanna Szaefer, Barbara Licznerska, Hanna Sobierajska, Wanda Baer-Dubowska

**Affiliations:** Department of Pharmaceutical Biochemistry, Poznan University of Medical Sciences, 60-806 Poznań, Poland; barlicz@ump.edu.pl (B.L.); bernatha@ump.edu.pl (H.S.); baerw@ump.edu.pl (W.B.-D.)

**Keywords:** cytochrome P450, flavonoids, resveratrol and derivatives, glucosinolates breakdown products, breast cancer

## Abstract

Estrogens are considered the most important risk factor for the development of breast cancer. Therefore, attempts are being made to reduce their level through diminished synthesis on one hand and to protect against the formation of DNA-damaging estrogen metabolites on the other. Cytochromes P450 (CYPs) play key roles in estrogen synthesis and catabolism, leading to potentially carcinogenic metabolites. CYP19 (aromatase) catalyzes the conversion of androgens to estrogens. The estrogen receptor-dependent pathway induces cell growth. CYP1 family enzymes, particularly CYP1B1, are involved in the redox cycling of estrogen metabolites and the subsequent estrogen–DNA adducts formation. Naturally occurring phytochemicals of different classes were shown to modulate the CYP expression and activity in cell-free systems or breast cancer cells. One of the most promising CYP19 inhibitors is chrysin (flavone), while stilbenes seem to be the most effective CYP1B1 inhibitors. In most cases, their effect is not specific. Therefore, different approaches are made to find the best candidate for the drug prototype of a new therapeutic or chemopreventive agent and to improve its pharmacokinetic parameters. This review presents and discusses the possible effects on major CYPs involved in estrogen metabolism by phytochemicals from the most investigated classes, namely flavonoids, stilbenes, and glucosinolates breakdown products.

## 1. Introduction

Breast cancer, with the diagnosis of 2.3 million women in 2024, has been recognized as one of the most commonly diagnosed cancers worldwide [1]. Although mortality related to this type of cancer has been dropping since the 1990s [2], we must continue to reduce these numbers.

The estrogen-sensitive nature of breast cancer was observed by Beatson over 100 years ago [3], and still, estrogens are considered one of the main breast cancer risk factors. Estrogens act through estrogen receptor (ER)-mediated events and contribute to cancer development not only by promoting cell proliferation, changes in differentiation, and gene expression, but also by initiating carcinogenesis via reactive metabolites [4]. Estrogen generally acts through two estrogen receptors: ERα and ERβ. ERα is present in almost 50–80% of breast cancer cases, and its expression correlates with better prognosis and a lower risk of recurrence. ERβ has also been detected in breast tumors, and is suspected to contribute to hormonal sensitivity and resistance. ERα mediates most estrogen signaling in classic estrogen targets, while ERβ plays a minor role. Hence, ERα is considered the most important target of chemopreventive and therapeutic agents in breast cancer [5].

Therefore, intervention in estrogen synthesis resulting in a reduced estrogen level and its catabolism leading to the formation of reactive genotoxic metabolites are equally important in searching for chemopreventive or therapeutic agents, including phytochemicals. In both pathways, cytochromes P450 (CYPs) play a crucial role, catalyzing key steps, such as aromatization of androstenedione to estrone, testosterone to estradiol, or formation of hydroxylated estrogens, mainly estradiol metabolites. The latter are precursors of highly reactive semiquinones and quinones, which can damage DNA and form DNA adducts.

The outcome of pharmacological interventions is dependent on the breast cancer subtype. Immunohistochemical and molecular expression profiles of key genes studies have classified breast cancer into four broad subtypes: estrogen receptor-positive (ER+), progesterone receptor-positive (PR+), human epidermal growth factor receptor 2-positive (HER2+), and triple-negative [6]. Patients diagnosed with the latter subtype are classified as triple-negative breast cancer (TNBC), the most invasive and metastatic breast cancer with a poor prognosis and early recurrence rate [4]. TNBC lacks the ERα, but the expression of ERβ and G-protein-coupled estrogen receptor can trigger estrogen responsiveness in this type of breast cancer [7].

About 80% of breast cancer tumors are positive for the hormone receptors, wherein binding of the requisite chemical messenger to their receptor causes changes in the cancer cell resulting in proliferation [8]. These receptors can be targeted by drugs that disrupt the signaling process. However, treatment with these drugs is often associated with undesirable side effects [9]. Therefore, all breast cancer subtypes still need highly effective chemopreventive and therapeutic agents. Several naturally occurring phytochemicals can alter the biosynthesis and catabolism pathways of estrogens by affecting the CYPs that catalyze their key steps. Such activity makes them promising candidates for use as chemopreventive or therapeutic agents against breast cancer. Among them, flavonoids, sulfur-containing breakdown products of glucosinolates (GLSs), and stilbene derivatives are particularly noteworthy in this context. This review presents the activities of flavonoids (e.g., chrysin, naringenin, galangin, quercetin), resveratrol and its derivatives (e.g., pinostilbene, pterostilbene, 2,3′,4,5′-tetramethoxy-trans-stilbene), indole-3-carbinol, 3,3′-diindolomethane, sulforaphane, lignans, and lycopene on CYPs involved in breast cancer development. It provides arguments for their possible application as breast cancer chemoprevention and/or therapy.

## 2. Characteristics of the Major CYPs Involved in Estrogen Synthesis and Catabolism

The human cytochrome P450 (CYP) superfamily of enzymes belongs to the class of oxidoreductases and consists of 57 isoforms that can metabolize a vast variety of endogenous and xenobiotic compounds [10]. Although human CYPs are mainly expressed in the liver, their expression in extrahepatic tissues, including ovaries and breast epithelium, is also well documented [11]. Moreover, in the great majority of breast carcinoma cases in postmenopausal women, biologically active estrogens are locally produced in an intracrine mechanism in the breast cancer tissues, which is linked with increased activity of CYPs involved in estrogen synthesis [12]. Therefore, differential endogenous expression of certain CYP genes in tumor vs. normal tissues provides the opportunity for selective targeting with small molecules, including phytochemicals. In the case of breast cancer, CYPs involved both in estrogen synthesis and catabolism are potential targets for chemopreventive and/or therapeutic agents. Some, like orphan CYPs, might serve as biomarkers allowing monitoring of the therapy, but also therapeutic targets.

### 2.1. CYPs and Estrogen Biosynthesis

Elevated circulating estrogen levels are considered one of the main risk factors of breast cancer by promoting its development via proliferation stimulation, changes in differentiation, and gene expression. Therefore, intervention in the estrogen synthesis pathway represents a reasonable approach to reduce breast cancer risk.

**Figure 1:**
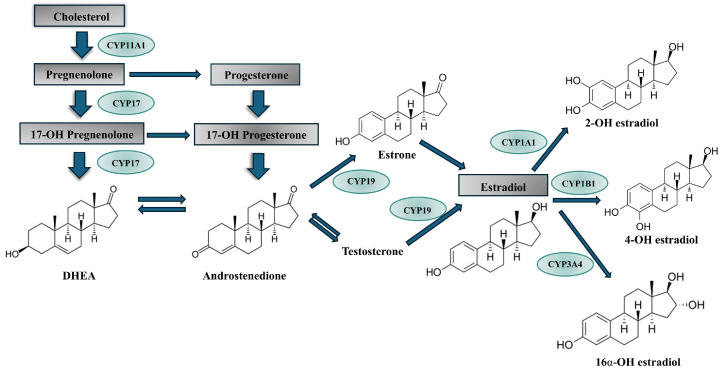
Cytochrome P450 isoforms involved in estrogen metabolism (chemical formulas were created with ChemSketch 2021.2.1).

The biosynthesis of estrogen from cholesterol involves several enzymatic steps, in which oxidation reactions by P450 enzymes play key roles (Figure 1). Among the five CYPs involved in steroid oxidation, three catalyze the reactions leading to the formation of estrogens.

CYP11A1 catalyzes the formation of pregnenolone from cholesterol. CYP17A1 is involved in two possible pathways of the formation of androgens, and CYP19A1 catalyzes the formation of estradiol [13]. In premenopausal women, the ovaries and adrenal glands are the principal source of estradiol. In postmenopausal women, the circulating inactive steroids such as androstane, testosterone, and estrone sulfate are thought to be major precursor substrates of local estrogen production [14]. CYP11A1 is the classical cholesterol side chain cleavage enzyme that converts the sterol cholesterol into other steroids. It also uses other sterols as substrates, as well as vitamin D [15,16].

CYP17A1 is a critical enzyme in the production of androgens, which are involved in prostate cancer development. The enzyme catalyzes two reactions: the 17-α-hydroxylation of both progesterone and pregnenolone and the subsequent “lyase” reaction to generate the androgens and androstenedione and dehydroepiandrosterone (DHEA), respectively [11]. Most drugs that target CYP17A1 in prostate cancer treatment inhibit both reactions, but the product of the first reaction is needed for the synthesis of glucocorticoids. Therefore, searches for selective inhibitors of the lyase step are ongoing [17].

While the above CYPs catalyze intermediate reactions in the synthesis of all steroids, CYP19A1 is specifically linked to estrogen synthesis. This enzyme converts androgens into estrogens [13]. Although CYP19A1 is encoded by a single gene, differential splicing results in the production of tissue-specific proteins. A reduced level of CYP19A1 diminishes androgen synthesis but is beneficial in treating female endocrine cancers. Therefore, inhibition of CYP19A1 has become an established therapeutic strategy for these types of cancers [18,19,20].

### 2.2. CYP-Mediating Estrogen Catabolism

Estrogens are metabolized mainly in the liver to a large number of hydroxylated products, which include 2-, 4-, 6α-, 6β-, 7α-, 12β-, 15α-, 15β-, 16α-, and 16β-hydroxylated metabolites. Among them, 2- and 4-hydroxyestradiol can be subsequently *O*-methylated to monomethoxy estradiol metabolites by catechol *O*-methyltransferase (COMT). While 2-methoxyestradiol appears to be non-carcinogenic and inhibits the proliferation of cancer cells, 4-hydroxyestradiol undergoes metabolic redox cycling to generate free radicals such as superoxide and the reactive semiquinone/quinone intermediates, causing DNA damage, which may lead to the initiation of the carcinogenesis process [21].

In the formation of hydroxylated estrogen metabolites, mainly CYP1A1, CYP1A2, CYP3A4, and CYP1B1 isoforms are involved. While CYP1A1, CYP1A2, and CYP3A4 exhibit catalytic activity in the 2-hydroxylation, CYP1B1 is responsible for the 4-hydroxylation of estradiol [22]. The latter reaction is predominant in the human breast where the expression of CYP1B1 is high [23]. Minor metabolites of estradiol are also produced in reactions catalyzed by the other CYPs such as CYP1A2, CYP2A6, CYP2C8, CYP3A4, and CYP3A7 [22].

The expression of CYP1B1 is abundant in tumor tissues [24]. This observation strongly suggested that the specific and local formation of 4-hydroxylation of estradiol is important for breast and endometrial carcinogenesis, and implicates CYP1B1 as a key player in this process. However, there have been conflicting reports regarding the expression of CYP1B1 in tumors vs. the adjacent non-tumor mammary gland tissues [25]. One reason for this discrepancy could be the increased sensitivity of transcript detection compared to protein. In addition, it should be considered that transcript, and protein levels might not reflect the actual enzymatic activity. The lower expression of the CYP1B1 gene in cancerous tissue relative to adjacent tissue could also be attributed to the possible downregulation of the gene by the AhR repressor in the tumor or by methylation of the promoter region, resulting in epigenetic silencing [26]. Nonetheless, expression of CYP1B1 in hormone-mediated malignancies is acknowledged to be important in the regulation of progression, metabolism, treatment, and resistance of breast tumors [23].

CYP1A1/A2 and CYP1B1 are induced through the binding of substrate to the cytosolic AhR. Crosstalk between AhR and ER signaling is well documented, but the mechanisms of reciprocal AhR–ER interactions have not yet been entirely explained. While, in most cases, the inhibitory effects of AhR on ERα were observed, the AhR-mediated estrogenic effects were also described [27]. Therefore, the complexity of crosstalk between AhR and ERα requires consideration in searching for new chemopreventive or therapeutic agents, particularly for treating hormone-dependent cancers such as breast cancer.

### 2.3. Orphan CYPs and Breast Cancer

It is estimated that of the 57 human cytochromes P450 (P450) and 58 pseudogenes discovered to date, 1/4 remain “orphans” in the sense that their functional expression sites and regulation are still largely not elucidated [28]. Several orphan CYPs were detected in breast epithelium. Although most of them seem to be expressed in different cancer tissues, one, namely CYP4Z1, is considered specific for the breast epithelium. The currently available data indicate that inhibition of CYP4Z1 breast-specific expression may reduce the growth, progression, angiogenesis, and invasiveness of breast cancer [29,30]. CYP4Z1 facilitates breast cancer development by induction of ERα expression; thus, its inhibition may have a double effect, eliminating the major breast cancer risk factor. Therefore, CYP4Z1 may slow down tumorigenesis initiation and prevent tumor development.

Other orphan CYPs, such as CYP2W1, CYP2S1, CYP2U1, and CYP4X1, although not as specific for breast epithelium as CYP4Z1 is, show much higher expression in tumors than in normal tissues [31]. Most of them seem to catalyze fatty acid hydroxylation, leading to the production of signaling molecules such as epoxyeicosatrienoic acids (EETs) or hydroxyeicosatetraenoic acid (HETE). Both are considered critical modulators of cancer progression involved in promoting cellular proliferation, neovascularization, angiogenesis, and metastasis. Therefore, inhibition of the expression and activity of these orphan CYPs might be more useful in cancer treatment than prophylaxis [32,33,34,35].

### 2.4. Structures of Major CYPs Involved in Estrogen Synthesis and Catabolism

All CYPs are characterized by a heme-iron center in the active site, tethered by a cysteine thiolate ligand localized in a characteristic FXXGXXXCXG element in their amino acid sequence. The shared tertiary structures usually contain 12 common helices (A-L) and four common β-sheets. Although the size and shape of various substrates for CYPs are diverse, they usually enter the active site near the connection between the F and G helices [36].

The determination of the crystal structure of active CYP19A1 purified from human placenta in complex with its natural substrate androstenedione in the high-spin ferric state of heme indicated hydrogen bond-forming interactions and tight packing of hydrophobic side chains that closely complement the puckering of the steroid backbone. This structure provides the molecular basis for the androgenic specificity of aromatase. Moreover, in the crystal, aromatase molecules are linked by a head-to-tail intermolecular interaction via a surface loop between helix D and helix E of one aromatase molecule that penetrates the heme-proximal cavity of the next molecule, thereby forming a polymeric aromatase chain in tandem. This intermolecular interaction is driven by electrostatics between the negatively charged surface of the D–E loop region and the positively charged heme-proximal cavity. There are only a few examples of similar head-to-tail oligomerization in the P450 structure database [37]. Furthermore, studies using an amino-terminus-truncated recombinant human aromatase showed that catalytic amino acids at the heme distal site are close to the bound steroid and may directly participate in the reaction mechanism. The amino acids at the active site access channel are likely responsible for the passage of water and steroids, as well as proton flow [38]. All data on CYP19 structure, including 3D and function, are deposited with the Protein Data Bank (e.g., entry 4KQ8 or 5jkv).

As mentioned in the previous subsection, CYP1A1 and CYP1A2 primarily catalyze the 2-hydroxylation of estradiol, while CYP1B1 predominantly catalyzes the 4-hydroxylation of estradiol. Homology modeling of the three-dimensional structures of CYP1A1 and CYP1B1 using the crystal structure of CYP1A2, along with the study of estradiol’s docking mode with CYP1A1, CYP1A2, and CYP1B1, demonstrated that two specific amino acid residues for each CYP—namely Thr124 and Phe260 in CYP1A2, Ser122 and Phe258 in CYP1A1, and Ala133 and Asn265 in CYP1B1—are important for estradiol recognition [39]. The RCSB Protein Data Bank provides experimentally determined 3D structures for CYP1B1 (e.g., 61Q5) and for CYP1A1 and CYP1A2 (e.g., 605Y and 2HI4, respectively).

## 3. Targeting Breast Cancer Cytochromes P50 by Naturally Occurring and Modified Phytochemicals

### 3.1. Flavonoids

**Figure 2:**
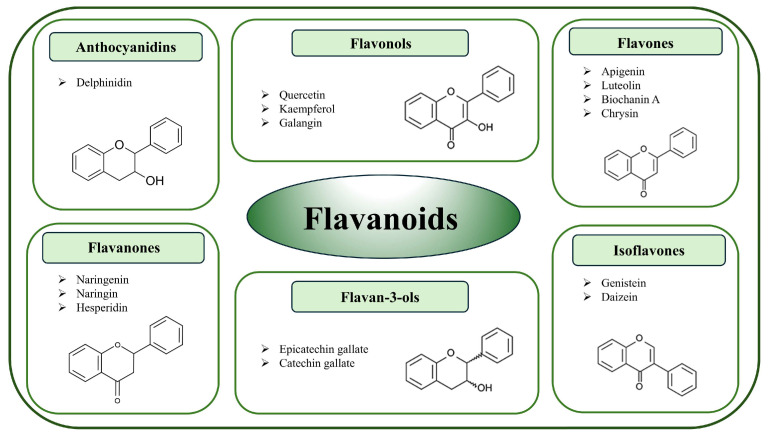
Classification of flavonoids with the examples of each subgroup (chemical formulas were created with ChemSketch).

Flavonoids constitute the largest and most diverse group of plant polyphenols. Based on the chemical configuration of hydroxy groups and degree of oxidation, flavonoids are classified into six subgroups: flavanones, flavones, isoflavones, flavonols, flavononol, and anthocyanins with proanthocyanidins (Figure 2) [40,41].

Among them, isoflavones are a particularly interesting class of flavonoids since they are structurally very similar to estrogen and elicit weak estrogenic and antiestrogenic effects [42,43]. Due to their similarity to estrogen and their estrogenic activity, isoflavones are classified as phytoestrogens. The three most abundant isoflavones are genistein, daidzein, and glycitein, found mainly in leguminous plants such as soy [44]. While most of the flavonoids are glycosylated, flavonols are not. Anthocyanins are compounds that are mainly concentrated in the skins of fruits and vegetables and are responsible for their red, purple, and blue colors. They exist principally as glycosides called anthocyanidins [44]. Anthocyanins have not, to date, been investigated for their effects on CYPs related to estrogens’ metabolism. The representatives of almost all groups of flavonoids showed the capacity to affect breast cancer cells in preclinical models. Moreover, epidemiological studies mainly referring to the Mediterranean diet and some clinical trials confirmed the association of flavonoid consumption with reduced breast cancer incidence [45].

Different mechanisms leading to limiting breast cancer cells’ proliferation or death by flavonoids were described and include modulation of CYPs involved in estrogen synthesis and catabolism. It is possible that the most extensive effect on CYP19 (aromatase) expression and activity was investigated. In this regard, a comprehensive review by Adam and Chen in the year 2009 [40] summarized the data on aromatase inhibitors among this class of phytochemicals obtained mainly in cell-free systems. A more recent review by Seth et al., along with these data, presents more extensive information obtained on breast cancer cells’ cultures and drives the conclusion that flavonoids can inhibit aromatase and thus prevent estrogen binding to ERα through the inactivation of aromatase downstream pathways and ultimately lowering cancer cell proliferation [46].

The largest group of flavonoids investigated as aromatase inhibitors is flavones. In this regard, early studies of Kellis and Vickery [47] using placental microsomes showed that among natural flavones tested, the most effective were chrysin, followed by apigenin. In a similar system, the other authors tested baicalein and galangin, and 7,8-dihydroxyflavone. While baicalein did not inhibit the aromatase activity, the highest activity of chrysin was confirmed. Moreover, the site-directed mutagenesis study suggested that the hydroxyl group at C-5 plays an important role in binding flavones to the enzyme [48].

In most cases, flavones were competitive inhibitors of aromatase, although an allosteric mechanism of inhibition was also suggested [47]. The anti-aromatase activity of flavones, particularly chrysin, apigenin, and biochanin A, was confirmed in cell systems. For instance, in the co-culture of MCF7 breast cancer cells with fibroblasts that mimic the interaction between tumor cells and surrounding tissue, all these flavones inhibited aromatase activity, with chrysin being the most effective [49]. The high efficacy of chrysin in inhibiting CYP19 was corroborated in several subsequent studies summarized in the review by Balam et al. [50]. The anti-aromatase activity of flavonols was assessed similarly to that of flavones in human placental microsomes [51,52], as well as in preadipocyte and adrenocortical cell cultures. Catechin and epicatechin did not exhibit anti-aromatase activity. However, catechin gallate, epigallocatechin gallate (EGCG), and gallocatechin gallate were shown to be strong aromatase inhibitors. Kinetic studies indicated a mixed mechanism of enzyme inhibition [40]. Among flavanones, hesperidin, 7-methoxyflavanone, and 4′-hydroxyflavanone have been identified as aromatase inhibitors [46]. In this regard, Li et al. [53] showed that luteolin also decreased the level of CYP19 transcript in MCF7 cells. The anti-aromatase activity of naringenin varied based on the inhibitory assay. The flavonol quercetin glycoside, along with the glycosides of kaempferol, fistin, and morin, either did not affect aromatase activity or exhibited mild effects similar to kaempferol [40]. Additionally, isoflavones such as daidzein and genistein, which demonstrate both estrogenic and anti-estrogenic activities, showed a mild effect on aromatase, with inconsistent data [46]. However, although glyceollin does not inhibit aromatase, it may help overcome resistance to the standard anti-aromatase drug, letrozole [54]. A more recent study on the anti-aromatase activity of flavonoids from citrus peels, including naringenin, naringin, and quercetin, provided evidence of their enzyme inhibition and anticancer activity in vivo, as indicated by decreased tumor volume [55]. Moreover, the in vitro analysis of the panel of 14 flavonoids showed their potential in CYP19 inhibition. Next, the predicted binding affinities were assessed using in silico models [56].

Several studies evaluated the effects of different flavonoid subgroups on the enzymes involved in estrogen catabolism. As with the anti-aromatase investigations, most of these studies were conducted in vitro using cell-free systems or cell cultures. The latter encompassed various breast cancer cell lines and non-tumorigenic MCF10A cells. In this context, an early study by Ciolino et al. [57] assessed the effects of diosmine (a flavone glycoside) and its aglycone in MCF7 human breast epithelial cancer cells. Diosmetin, but not diosmine, directly inhibited CYP1A1 activity in a non-competitive manner in microsomes isolated from dimethylbenz[a]anthracene (DMBA, AhR agonist)-treated cells. However, in intact cells, an increase in CYP1A1 activity was observed, comparable to that induced by DMBA. Both compounds elevated the transcription of the CYP1A1 gene and activated the DNA-binding capacity of the AhR to the xenobiotic-responsive element of CYP1A1. These results suggested that diosmin and diosmetin act as agonists of AhR, leading to enhanced CYP1A1 transcription, but only diosmin is capable of inhibiting CYP1A1 enzyme activity and ultimately carcinogen activation. Since this CYP isoform catabolizes estrogen, it may be speculated that a similar mechanism applies to estrogen metabolism. Essentially, comparable data were provided by the same group regarding the flavonol galangin [58] and the flavonols quercetin and kaempferol [59].

Growing evidence points to a crucial role for CYP1B1 in cancer and other diseases through the impaired metabolism of endogenous compounds such as estrogens [60]. Several flavonoids were tested in this context and showed that some of them might be selective inhibitors of this CYP isoform. Takemura et al. [61] investigated the effect of methoxylated flavonoids on CYP1A1, 1A2, and CYP1B1 activity (ethoxyresorufin *O*-deethylation assay) and found that all flavonoids tested in this study selectively inhibited CYP1B1 activity. Methoxy types of flavones and flavonols, such as chrysoeriol and isohamnetin, showed strong and selective inhibition of CYP1B1. Molecular docking analysis indicated that methoxyflavonoids with 2–3 double bonds, chrysoerol, and isorhamnetin fit well into the active site of CYP1B1, but not into the active site of CYP1A2 and CYP1A1. Moreover, quercetin and glabrol, the components of *Glycyrrhiza glabra*, not only showed selective inhibition of CYP1B1 in breast cancer cells but also were able to reverse cisplatin resistance in CYP1B1 overexpressing MDA-MB-468 (TNBC) cells [62]. On the other hand, nobiletin and polymethoxyflavone were shown to induce their own metabolism, which may affect their cytostatic effect in MCF7 breast cancer cells, via CYP1A1 and CYP1B1 upregulation [63]. Similarly, increased expression of CYP1A1 and CYP1B1 in MCF7 breast cancer cells as a result of treatment with methoxylated flavones, eupatorine, and cirsiliol was observed. Moreover, it was demonstrated that cirsiliol is subsequently converted by CYP1B1 or CYP1A1 into an antiproliferative agent [64]. A more recent study of the effect of another polymethoxylated flavone, tangeretin, showed its conversion to 4′-OH tangeretin by recombinant CYP1 enzymes expressed in ER(+) MCF7 and MDA-MB-468 (TNBC) cells. This metabolite was absent in non-tumorigenic MCF10A cells. The latter characterized the low expression of CYP1 enzymes in non-cancer cells [65]. Tangeretin was subsequently shown to induce CYP1 activity and CYP1A1/CYP1B1 expression in MCF7 and MDA-MB-468 cells, suggesting that this flavone inhibits the proliferation of breast cancer cells via CYP1A1/CYP1B1-mediated metabolism to 4′-hydroxy tangeretin [66]. The metabolism of hydroxylated flavones and flavonols, apigenin, luteolin, scutellarein, kaemferol, and quercetin in similar models, i.e., CYP1 recombinant enzymes and MCF7, MDA-MB-468, and MCF10A cell lines was analyzed. In addition, molecular docking was performed, indicating that CYP1B1 favored the A ring orientation of apigenin and luteolin to the heme group compared with CYP1A1 [67]. The assessment of cytotoxicity in the tested cell lines pointed out luteolin as the most potent in TNBC MDA-MB-468 cells. Luteolin metabolism yielded 6-hydroxyluteolin only by recombinant CYP1B1, suggesting that this metabolite might be responsible for enhancing the antiproliferative activity of this flavonoid. While the flavonoids described above were metabolized mainly by CYP1A1 and CYP1B1, isoflavone daidzein and genistein were metabolized by CYP1A2 in cell-free systems (liver preparations). The effects of daidzein metabolites 6,7,4′-trihydroxyisoflavone and 7,3′,4′-trihydroxyisoflavone were studied in MCF7 cells. The latter metabolite reduced total cell numbers to a greater extent than 6,7,4′-trihydroxyisoflavone or daidzein and increased cell death [68]. On the other hand, the study of the effect of genistein and daidzein on the expression of E2 metabolizing enzymes in MCF7 cells showed that both phytoestrogens at the estrogen-active concentrations stimulate the formation of the more genotoxic metabolites and inhibit the detoxication of catechol and quinone estrogens in these ER(+) cells [69]. However, in non-tumorigenic breast cells, the MCF10A line, genistein protected against PAH-induced oxidative damage most probably by affecting CYP1A1 and CYP1B1 expression or activity [70].

To sum up, flavonoids of different structural groups affect CYPs involved in estrogen synthesis and catabolism. In general, flavones are the most active and most intensively studied. Most data concerning their activity were obtained in cell-free systems or cell cultures, and often discrepancy is observed in the results obtained in these two systems. Many representatives of the group of phytochemicals are metabolized by CYP1 family enzymes. Therefore, still more profound studies on their application in breast cancer prevention or treatment are needed.

### 3.2. Resveratrol and Its Derivatives

A naturally occurring phytoalexin, *trans*-resveratrol (3,5,4′-trihydroxy-trans-stilbene), is present in numerous plants, but it is most commonly found in red grape skin. Jang et al. originally reported in 1997 the inhibitory effect of resveratrol on all stages of carcinogenesis in the mouse skin model and the development of preneoplastic lesions in carcinogen-treated mouse mammary glands [71]. Subsequently, numerous studies have demonstrated its anti-tumorigenic activity in vitro, including the human breast cancer cells, and in vivo models [72,73,74]. Several reports have demonstrated that resveratrol inhibits the expression of many cytochrome P450 genes, including those encoding CYP1A1, CYP1B1, CYP1A2, and CYP19 in cancer cell lines of different tissue origin in humans. It has been suggested that this inhibition, at least in part, may underlie the anticancer effects of this compound [75,76,77].

Resveratrol is a polyphenolic compound that has structural similarity to estrogen. This nonflavonoid phytoestrogen inhibited CYP19 (aromatase) activity in numerous experimental breast cancer models, including MCF7aro cells overexpressing the *CYP19* gene [78]. Also, in the study conducted by Khan et al. [18] using SK-BR-3 breast cancer cells (cell line overexpressing the *HER2* gene), resveratrol significantly reduced the expression and level of CYP19 protein in a dose-dependent manner. Moreover, this compound was able to repress the transactivation of CYP19 promoters I.3 and II in SK-BR-3 cells, which indicates that resveratrol could be able to reduce localized estrogen production in breast cancer cells [18]. In ER(+) T47D-BAF cells co-cultured with breast adipose fibroblasts, resveratrol showed an aromatase inhibitory effect with a potency comparable to letrozole, which is a clinically used anti-aromatase drug in breast cancer treatment [79]. Our research, conducted on ER(+) MCF7 and ER(−) MDA-MB-231 breast cancer cells and non-tumorigenic breast MCF10A cells, has shown that resveratrol modulated the expression of CYP19. In this regard, we have shown that resveratrol reduced the level of *CYP19* transcript and protein level in both MCF7 and MDA-MB-231 breast cancer cells. However, the opposite effect was noted in MCF10A breast cells [80,81].

Numerous studies focused on the effect of resveratrol on the modulation of CYP1A1 and CYP1B1 involved in estrogen catabolism. Many of these studies have demonstrated that resveratrol inhibits dioxin induction of CYP1A1 and CYP1B1 in a dose-dependent manner. There have been a number of contradictory observations regarding the mechanism of this inhibitory effect, and no unified model has arisen [75,82,83]. In this regard, Beedanagari et al. [84], based on the results obtained in MCF7 breast cancer cells in which resveratrol significantly reduced dioxin-induced CYP1A1 expression, suggested that the resveratrol mechanism of action involves inhibition of the recruitment of transcription factors AhR and ARNT recruitment to the xenobiotic response elements (XREs) of the corresponding genes. As a result, the binding of polymerase II to the promoter CYP1 family genes is reduced. The studies conducted by Lee and Safe [83] confirmed the inhibition of 2,3,7,8-tetrachlorodibenzo-p-dioxin (TCDD) induced CYP1A1 activity and expression by resveratrol at a dose of 10 μM in MCF7 and T47D breast cancer cell lines. Another study in a non-tumorigenic mammary gland cell line, MCF10F, demonstrated that resveratrol reduced TCDD-induced CYP1B1 expression in a dose-dependent manner. Moreover, CYP1B1 expression was almost completely inhibited by resveratrol [85]. Resveratrol also inhibited TCDD-induced CYP1A1 and CYP1B1 expression levels and recruitment of AhR in MDA-MB-231 and BT-549 (TNBC) cells [86]. However, in vitro electromobility shift assay (EMSA) showed resveratrol triggering AhR to bind an XRE-containing double-stranded oligonucleotide, but resveratrol did not inhibit the TCDD-stimulated binding of AhR to the XRE sequence [83,87]. In contrast, other investigators, also using the EMSA, reported that resveratrol did not induce binding of AhR to an XRE-containing oligonucleotide, but did inhibit TCDD-induced binding of AhR to the XRE [75,82]. Interestingly, the inhibitory effect of resveratrol on DMBA-induced CYP1A1 expression was observed in the non-tumorigenic breast MCF10A cells [88]. Our research performed on MCF7 and MDA-MB-231 breast cancer cell lines has shown that resveratrol inhibited the expression of CYP1B1 [81]. In the non-tumorigenic breast MCF10A cells, the inhibitory effect of expression was observed in the case of both CYP1A1 and CYP1B1 [80]. The inhibitory effect of resveratrol in the breast cancer cell model was also confirmed in our other studies in the context of modulation of orphan cytochromes. In ER(−) MDA-MB-231 cells, resveratrol reduced the protein level of two orphan cytochromes: CYP2W1 and CYP2S1 [89]. Therefore, it can be concluded that resveratrol affects key enzymes involved in estrogen synthesis and catabolism. The ultimate effect is dependent on the dose and cell types.

Although resveratrol has a broad spectrum of anticancer activity, its fast metabolism, low solubility in water, and low bioavailability limit its application [27]. Adding hydroxyl or methoxy groups to the stilbene backbone of resveratrol may improve its effectiveness. Indeed, several studies have indicated that such modifications increase resveratrol anticancer potency [80,90,91,92]. In the study conducted by van den Brand et al. [93], the anti-tumor properties of resveratrol along with its natural and synthetic analogs, oxyresveratrol, pinostilbene, pterostilbene, and 2,3′,4,5′-tetramethoxy-trans-stilbene (TMS) were tested in MCF7 breast cancer cells and non-tumorigenic MCF10A cells. Assessment of cell viability and migration showed that methoxylated analogs of resveratrol are more potent antitumorigenic compounds than resveratrol and its hydroxylated analog oxyresveratrol. Moreover, TMS was the most potent compound in this regard. Additionally, TMS increased AhR-mediated induction of CYP1A1 activity more than resveratrol or the other analogs. Induction of CYP1A1 expression means that active estrogen molecules will be metabolized more rapidly into 2-hydroxy derivatives that lack estrogenic or procarcinogenic activity. Therefore, this effect of TMS is beneficial, especially since it is more potent than the effect of resveratrol itself, whose more stable and more active analogues we are seeking. Our studies revealed that naturally occurring *trans*-resveratrol analogs, pinostilbene (3,4′-dihydroxy-5-methoxystilbene), desoxyrhapontigenin (3,5-dihydroxy-4′-methoxystilbene), and pterostilbene (3,5-dimethoxy-4′-hydroxystilbene) inhibited human recombinant CYP1A1 more effectively than the parent compound. In contrast, pterostilbene, deoxyrapontigenin, and pinostilbene affected CYP1B1 similarly to resveratrol [94].

In our other studies, *trans*-stilbene compounds with tri-, tetra-, and penta-methoxy groups were studied in non-tumorigenic breast epithelial cells (MCF10A) and breast cancer cells (MCF7 and MDA-MB-231). Mostly, the decreased expression of CYP1A1 and CYP1B1 was observed following the administration of these chemicals in MCF10A cells. 3,4,2′,4′,6′-pentamethoxy (5MS) was the most effective inhibitor of the expression of the CYP1A1 and CYP1B1 genes. In the case of breast cancer cells, the CYP1A1 protein level was slightly reduced by 3,4,2′-trimethoxy (3MS) and 3,4,2′,4′-tetramethoxy (4MS), and the CYP1B1 expression was decreased as a result of the treatment with 4MS, but only at the transcript level in MDA-MB-231 cells [80,81].

Chun et al. [95] indicated that another 2,4,3′,5′-tetramethoxystilbene (TMS), a synthetic trans-stilbene analog, significantly inhibited TCDD-stimulated CYP1B1 protein and mRNA expression in a concentration-dependent manner in MCF7 and MCF10A cells. TMS acts as a strong modulator of CYP1B1 gene expression as well as a potent selective inhibitor in vitro [60].

**Figure 3:**
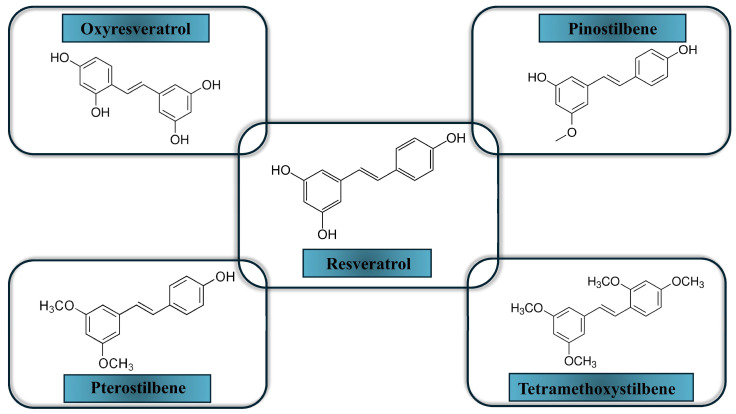
Resveratrol and its chosen derivatives (chemical formulas were created with ChemSketch).

These results indicate that the methoxy *trans*-resveratrol analogs may be more effective inhibitors of CYPs involved in estrogen catabolism than the parent compound. The final effect is dependent on the number of methoxy groups added to the stilbene structure and target cells [27]. So far, no attempt has been made to evaluate *trans*-resveratrol analogs in CYP19 expression and/or activity. Figure 3 shows the chosen resveratrol derivatives.

### 3.3. Glucosinolates Breakdown Products

**Figure 4:**
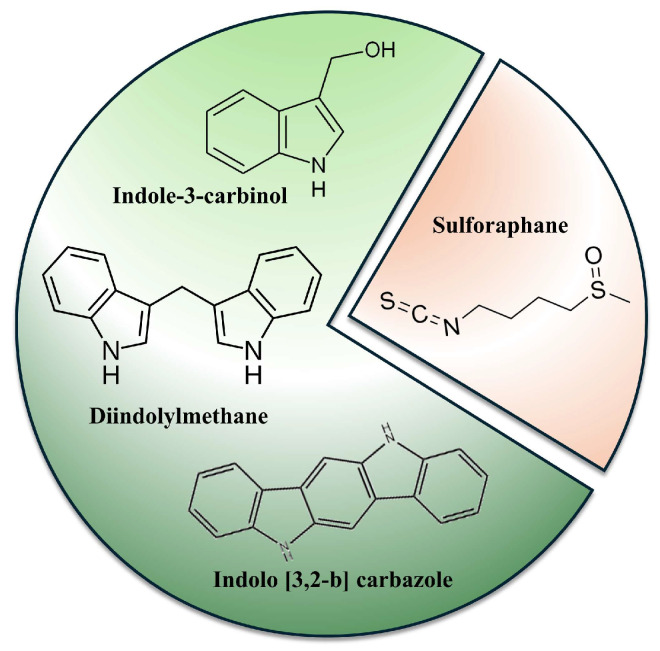
Structure of basic glucosinolate breakdown products (chemical formulas were created with ChemSketch).

Glucosinolates (GLS), such as glucobrassicin and glucoraphanin, are common ingredients of the Brassica vegetables, the members of the Brassicaceae family, also known as Cruciferae. These compounds contain sulfur and a common glycone moiety with variable side chains derived from amino acids. Disruption of plant tissue activates endogenous β-thioglucoside glucohydrolase (myrosinase), which catalyzes the GLS degradation, initially to unstable thiohydroximate-*O*-sulfonate, spontaneously rearranging to isothiocyanates (ITCs) or indoles [96]. The latter include indole-3-carbinol (I3C), 3,3′-diindolomethane (DIM), or indolo[3,2-b]carbazole (ICZ) (Figure 4). DIM is a product of I3C dimerization in an acidic environment, such as that in the stomach. The most interesting representative of ITC in the context of breast cancer prevention and/or therapy is sulforaphane (SFN) (Figure 4). The preventive efficacy of GLS degradation products, particularly indoles, against breast cancer, first observed in animal models, was confirmed in numerous mechanistic studies using cell cultures and supported by epidemiological studies involving cruciferous vegetables and their extracts or juices [96,97].

One of the possible mechanisms of this protective activity is the modulation of CYPs involved in estrogen synthesis and catabolism. In this regard, I3C and DIM reduced the expression of the CYP19 gene in breast cancer MCF7 cells [98]. In the case of SFN in ER(+) MCF7 cells, a significant reduction in CYP19 expression was observed, while in ER(−) MDA-MB-231, increased expression was noted. In non-tumorigenic MCF10A cells, the expression of CYP19 was not changed as a result of SFN treatment. Therefore, the possible application of SFN may be suggested as a therapeutic agent rather than a chemopreventive one [99].

There are also some interesting, although not numerous, reports on synthetic I3C derivatives that are CYP19 modulators. For example, De Santi et al. [100] showed that CTet (cyclic tetramer of I3C, synthetic derivative of I3C) inhibits testosterone aromatization in overexpressed CYP19 MCF7 cells. This finding may pave the way for searching for new aromatase inhibitors based on the indole scaffold.

The induction of CYP1, involved in estrogen catabolism, occurs upon activation of the AhR signaling pathway. As was delineated in the introductory section, estrogen metabolism catalyzed by CYP1A1 and CYP1B1 leads to the formation of reactive intermediates, such as E2–4-hydroxy derivatives and quinones, which bind to DNA, forming DNA adducts: the important event in tumor initiation. I3C, in contrast to DIM, is known as a strong AhR agonist [101]. Therefore, the early studies pointed out DIM as a markedly less efficacious inducer of CYP1 in rat models [102]. The analysis of metabolic profiles in these early studies indicated an increased metabolism of food-derived carcinogens through the induction of CYP1A1 and CYP1A2 by I3C [103]. On the other hand, in some systems, such as T47-D breast cancer cells, both I3C and DIM reduced CYP1A1 activity and expression induced by co-treatment with TCDD [104].

Our studies have shown the induction of CYP1A1 and CYP1B1 in different breast epithelial cell lines, namely ER(+) MCF7, ER(−) MDA-MB-231, and non-tumorigenic MCF10A, treated with both indoles [98]. However, the ratio of CYP1A1 to CYP1B1 was 1.22- to 10.6-fold in favor of CYP1A1 in MCF7 and MCF10A cells. Increased levels of CYP1A2 in comparison with CYP1B1 were also observed in MCF7 cells. In contrast, in MDA-MB-231 cells, CYP1B1 was preferentially induced. Taking into consideration the role of this isoform in breast cancer tissue, these observations confirmed the postulated ambiguous role of these indoles, particularly I3C, in cancer development [105]. CYP1A1 induction correlated with the 2-hydroxylation pathway by I3C in MCF7 breast cancer cells, but not in MDA-MB-231 cells, as also found in an earlier study by Ashok et al. [106].

The effects of ITCs on the expression and activity of CYPs involved in estrogen metabolism are ambiguous. SFN reduced CYP1A1 protein level equally in nontumorigenic MCF10A breast cells, ER(+) MCF7, and ER(−) MDA-MB-231 breast cancer cells, but the increased level of CYP1A2 and the decreased level of CYP1B1 expression were found only in MCF10A cells [99]. In addition, SFN and its analogs (isothiocyanate 2-oxohexyl and alyssin) inhibited CYP1A1 and CYP1A2 enzyme activity induced by the polycyclic aromatic hydrocarbons in MCF7 cells [107].

AhR and ER exhibit “cross-talk” through several mechanisms; the net results of GLS breakdown products, particularly I3C/DIM-dependent AhR modification of E2 metabolism, are a decrease in the ratio of 16α/2-hydroxy-E2 and a concomitant decrease in E2 levels. 16α-Hydroxy-E2 retains estrogenic activity and has been referred to as the procarcinogenic E2 metabolite, whereas 2-hydroxy-E2 has been termed the anticarcinogenic E2 metabolite. The ratio of 16α/2-hydroxy-E2 and total E2 levels are often used as a biomarker assessment of potential chemopreventive agents against estrogen-driven cancers [27,108,109]. In this regard, in the phase I study by Reed et al. [110], a maximal increase in the urinary 16α/2-hydroxy-E2 ratio was observed at a daily dose of 400 mg I3C, with no further increase observed at a dose of 800 mg/day. Besides confirmation of the optimal dose of I3C, these studies showed the induction of CYP1A2, which was mirrored by the increase in the 2-hydroxyestrone to 16α-hydroxyestrone ratio.

To summarize, the effects of GLS breakdown products on CYP enzymes involved in estrogen synthesis and catabolism are ambiguous. Its most notable aspect appears to be their ability, especially I3C/DIM-dependent AhR modification of E2 metabolism, which leads to lower levels of E2. Finding more selective inhibitors of CYP19 and CYP1B1, particularly those based on the indole scaffold, could help clarify the activity of these compounds.

### 3.4. Other Phytochemicals with Breast CYP Modification Potential

Lignans are plant compounds metabolized in the mammalian gut to produce the phytoestrogen metabolites enterolactone and enterodiol. The CYP17 genotype modifies the protective effect of lignans on the risk of breast cancer in premenopausal women. These results suggest that the CYP17 genotype may be important in modifying the effect on breast cancer risk of exogenous estrogens, particularly for premenopausal women [111,112]. Mechanistic/kinetic analysis showed that lignan enterolactone significantly decreased the amount of estradiol produced from androstenedione, indicating decreased aromatase (product of *CYP19* expression) activity in MCF7 cells [113].

Moreover, gomisin C and gomisin G, two lignan analogs derived from *Schisandra chinensis*, inhibited human CYP3A4 and CYP3A5 [114]. CYP3A4 catalyzes 16α-hydroxylation of estrogens and is involved in the metabolism of drugs used in breast cancer treatment regimens, e.g., catalyzes the conversion of TAM to the active 4-OH metabolite [115]. In addition, women with higher levels of 16α-hydroxy estrone in their urine are likely to have an increased risk of breast cancer [116]. Some clinical trials concluded that a diet including flaxseed (a rich source of lignans) was associated with increased levels of urine excretion of 2-hydroxy estrone, with 16α-hydroxy estrone levels unchanged [117].

In addition, the latest high-throughput sequencing and bioinformatics analysis revealed that miR-148a-5p expression was significantly up-regulated, whereas CYP1B1 expression was down-regulated following Honokiol, a lignin extracted from the *Magnolia* genus plant, treatment. High miR-148a-5p expression, analyzed with the TCGA database, showed a correlation with a better prognosis for breast cancer patients [118].

Lycopene, a tetraterpene carotenoid, found in high concentrations in red fruit and vegetables, inhibited recombinant CYP1A1 and CYP1B1, while in MCF7 cells, lycopene administration slightly reduced the DMBA-induced ethoxyresorufin-*O*-deethylase activity [119]. These data require confirmation in more extended studies.

## 4. Conclusions and Future Perspectives

Cytochromes P450 play a key role in estrogen-driven cancers, particularly breast cancer. Therefore, they are natural candidates for chemopreventive and/or therapeutic targets. While CYP19 might be considered exclusive for estrogen synthesis, the other CYPs, particularly CYP1 family enzymes, catalyzing their catabolism, are equally involved in the transformation of a plethora of exogenous compounds that might result in reciprocal effects.

Phytochemicals from various classes can modulate their expression and activity and, although not highly selective, have been extensively studied, particularly for inhibitors of CYP19 and CYP1B1 (Table 1). Inhibitors of the latter are highly desired since CYP1B1 catalyzes the formation of 4-hydroxy derivatives of estradiol, which initiates tumorigenesis. Its elevated levels in breast cancer tissue also make it a therapeutic target. Among the potential inhibitors of CYP19, the subgroup of naturally occurring flavones appears to be the most promising, with chrysin as a leading compound. Several inhibitors of CYP1B1 have been identified among various classes of phytochemicals, but almost all exhibit inhibitory activity toward other members of the CYP1 family, particularly CYP1A1, responsible for forming “good” estradiol metabolites. Synthetic modifications of natural phytochemical scaffolds may enhance their selectivity. An example is 2,4,3′,5′-tetramethoxystilbene (TMS), which demonstrated 50-fold selectivity for CYP1B1 over CYP1A1 and 500-fold selectivity for CYP1B1 over CYP1A2. Similarly, methylation of flavones has been shown to improve bioavailability and reduce side effects. Thus far, these positive effects have primarily been observed in mechanistic studies within cell-free systems. More preclinical investigations are needed to confirm their efficacy.

Phytochemicals interfering with estrogen metabolism may be useful not only as a potential therapeutic prototype but also in breast cancer adjuvant therapy to overcome resistance to clinically used drugs. Moreover, CYP1B1 inhibitors may prevent chemotherapy-induced cardiotoxicity [60]. Future research leading to clinical trials should focus on these issues.

While most of the phytochemicals presented and discussed in this review showed the ability to affect CYPs involved in estrogen synthesis and metabolism, it is still difficult to identify their selectivity toward specific CYP isoforms. Therefore, synthetic modification, including TMS, is the solution that has to be further developed. Moreover, as the low bioavailability of natural phytochemicals poses a problem in their application, nanotechnological interventions could be considered as cancer therapeutic strategies using these compounds. In this regard, quercetin conjugated with silica nanoparticles inhibited the growth of MCF7 breast cancer cells more efficiently than quercetin itself [120].

Modulation of CYPs involved in estrogen metabolism by different classes of phytochemicals was also demonstrated in natural food matrices such as cabbage juice, a source of indoles, or grapes and grape seed extracts, a source of resveratrol, and several flavonoids [79,99]. Therefore, these products might be recommended for breast cancer prophylaxis.

## Figures and Tables

**Table 1:** Summary of the effects of phytochemicals described in the text on breast cancer cytochromes P450.

Cytochrome P450	Compound	Biological Effect	Experimental Model of Cells	References
CYP19	Chrysin	Inhibition	MCF7 with fibroblasts co-culture	[49]
	Naringenin	Inhibition	MCF7, T47D	[55]
	Naringin	Inhibition	MCF7, T47D	[55]
	Quercetin	Inhibition	MCF7, T47D	[55]
	Resveratrol	Inhibition	SK-BR-3	[79]
		Inhibition	MCF7, MDA-MB-231	[81]
		Induction	MCF10A	[80]
	Indole-3-carbinol	Inhibition	MCF7	[98]
	3,3′-diindolomethane	Inhibition	MCF7	[98]
	Sulforaphane	Inhibition	MCF7	[99]
		Induction	MDA-MB-231	[99]
CYP1A1	Galangin	Induction	MCF7	[58]
	Quercetin	Induction	MCF7	[59]
	Resveratrol	Inhibition	MCF7 treated with TCDD *	[84]
		Inhibition	MCF7 and T47D treated with TCDD	[83]
	Indole-3-carbinol	Induction	T47D treated with TCDD	[104]
	3,3′-diindolomethane	Induction	T47D treated with TCDD	[104]
	Indole-3-carbinol	Induction	MCF7, MDA-MB-231, MCF10A	[98]
	3,3′-diindolomethane	Induction	MCF7, MDA-MB-231, MCF10A	[98]
	Sulforaphane	Inhibition	MCF7, MDA-MB-231, MCF10A	[99]
CYP1B1	Quercetin	Inhibition	MDA-MB-468	[62]
	Eupatorin cirsiliol	Induction	MCF7	[64]
	Resveratrol	Inhibition	MCF10F treated with TCDD	[85]
		Inhibition	MCF7, MDA-MB-231	[81]
	2,4,3′,5′-tetramethoxystilbene	Inhibition	MCF7 and MF10A treated with TCDD	[95]
	Indole-3-carbinol	Induction	MCF7, MDA-MB-231, MCF10A	[98]
	3,3′-diindolomethane	Induction	MCF7, MDA-MB-231, MCF10A	[98]
	Sulforaphane	Inhibition	MCF10A	[99]
CYP2W1	Resveratrol	Inhibition	MDA-MB-231	[89]
CYP2S1	Resveratrol	Inhibition	MDA-MB-231	[89]
CYP17	Lignans	Inhibition	MCF7	[113]

* TCDD: 2,3,7,8-tetrachlorodibenzo-p-dioxin.

## Data Availability

No new data were created or analyzed in this study.

## Abbreviations

The following abbreviations are used in this manuscript:

AhR	aryl hydrocarbon receptor
COMT	catechol O-methyltransferase
DHEA	dehydroepiandrosterone
DIM	3,3′-diindolomethane
EETs	epoxyeicosatrienoic acids
ER(+)	estrogen receptor positive
GLS	glucosinolates
HETE	hydroxyeicosatetraenoic acid
HER2	human epidermal growth factor receptor 2 positive
I3C	indole-3-carbinol
ITCs	isothiocyanates
PR(+)	progesterone receptor positive
TCDD	2,3,7,8-tetrachlorodibenzo-p-dioxin
TMS	2,3′,4,5′- tetramethoxy-trans-stilbene
TNBC	triple negative breast cancer
XRE	xenobiotic response elements
